# Single-cell reveals age-dependent epithelial reprogramming and EMT vulnerability in THCA

**DOI:** 10.1530/ERC-26-0021

**Published:** 2026-06-08

**Authors:** Qiankun Zhang, Wei Pan, Xiaohua Gong, Qi Zhou

**Affiliations:** ^1^ Department of Nephrology, The Fifth Affiliated Hospital of Wenzhou Medical University; Lishui Central Hospital; Lishui Hospital of Zhejiang University, Lishui, China; ^2^ Department of Endocrinology and Metabolism, The First Affiliated Hospital of Wenzhou Medical University, Wenzhou, China

**Keywords:** thyroid cancer, single-cell RNA sequencing, epithelial–mesenchymal transition, *PHTF2*, *SNAI1*, tumor microenvironment

## Abstract

Thyroid cancer exhibits pronounced cellular heterogeneity, yet the mechanisms underlying epithelial–mesenchymal transition (EMT) and tumor microenvironment (TME) remodeling remain incompletely understood. In this study, single-cell RNA sequencing of 92,849 cells from 20 thyroid tissue samples derived from underage and adult patients was performed to systematically characterize immune and epithelial heterogeneity through cell type annotation, subclustering, and functional enrichment analyses. A total of 26 distinct cell clusters were identified, encompassing immune and stromal compartments, with notable age- and stage-specific differences in T cell, epithelial, and macrophage subsets. EMT-high tumors were characterized by stromal enrichment, immune suppression, and transcriptional activation of canonical EMT drivers, including TGFB1, SNAI1/2, TWIST1/2, and ZEB1/2. Functional validation demonstrated that shRNA-mediated knockdown of PHTF2 or SNAI1 in TPC-1 and KTC-1 cell lines significantly inhibited proliferation and clonogenic capacity, accompanied by upregulation of CDH1 and downregulation of multiple EMT-associated genes. Furthermore, EMT-high tumors exhibited distinct drug sensitivity profiles, including reduced responsiveness to conventional chemotherapeutics and increased sensitivity to agents such as camptothecin and dabrafenib. Collectively, these findings identify PHTF2 and SNAI1 as key regulators of EMT and tumor cell proliferation in thyroid cancer, and suggest that EMT-driven TME remodeling contributes to immune evasion and therapeutic resistance, thereby revealing EMT-associated vulnerabilities as potential targets for precision therapy.

## Introduction

Thyroid cancer is among the most common endocrine malignancies, with papillary thyroid cancer (PTC) accounting for approximately 85% of cases (1, 2). PTC is generally considered indolent, with a favorable 10-year survival rate exceeding 90% following conventional treatments such as surgery and radioactive iodine therapy (3). However, disease recurrence and metastasis remain significant clinical challenges, contributing to increased morbidity and mortality. Histologically, PTC encompasses over fifteen variants defined by qualitative or semi-quantitative criteria, which often lack specificity and complicate accurate diagnosis (4). At the molecular level, recurrent alterations in *BRAF*, *RAS*, and *RET*/*PTC* genes are common, yet overall mutation burdens are relatively low compared with other cancers, limiting mutation-based classification (5, 6). Bulk molecular analyses classify PTCs as *BRAF*-like or *RAS*-like subtypes, reflecting distinct signaling activities, dedifferentiation states, and aggressiveness (7). For example, *BRAF*-like PTCs typically show constitutive MAPK activation, whereas *RAS*-like PTCs exhibit concurrent MAPK and PI3K pathway activation (7, 8). Additional pathways, such as NF-κB, are frequently activated in *BRAF* V600E tumors, promoting proliferation and inhibiting apoptosis (9). While molecular profiling offers quantitative insights beyond morphology, bulk approaches cannot capture intra-tumoral heterogeneity, particularly within the tumor microenvironment (TME) (10).

The TME in PTC consists of diverse stromal and immune cells, including fibroblasts, endothelial cells, lymphocytes, and macrophages. The composition and abundance of these cells vary across patients and even within tumors, influencing tumor progression, immune evasion, and therapeutic response (11). Single-cell RNA sequencing (scRNA-seq) enables high-resolution characterization of tumor and stromal cell subtypes, revealing transcriptional heterogeneity and functional states (12). However, tissue dissociation in scRNA-seq sacrifices spatial context. Spatially resolved transcriptomics (SRT) addresses this limitation by capturing gene expression patterns *in situ*, although current technologies operate at the spot level, often representing mixed cell populations (13). Integrating scRNA-seq and SRT is therefore essential to link cellular identity with spatial organization, providing mechanistic insights into tumor morphology, progression, and metastasis (14, 15).

PTC exhibits notable age-dependent heterogeneity. Tumors in children and young adults (underage-PTC) are more aggressive than in adult patients (adult-PTC), presenting with larger sizes, earlier capsule invasion, and higher lymph node metastasis rates (16). While genomic alterations in tumor cells have been characterized, the role of the TME in mediating these age-specific differences remains unclear. Immune components, including T cells, macrophages, and natural killer (NK) cells, are increasingly recognized as key modulators of tumor behavior (17). For instance, the *BRAF* V600E mutation facilitates the recruitment of immunosuppressive myeloid-derived suppressor cells, M2 macrophages promote the expansion of regulatory T cell populations through METTL3 downregulation, and the depletion of CD3-CD16-CD56^bright^ NK cells is associated with advanced disease, collectively highlighting the immune dysregulation underlying PTC progression (18, 19, 20).

Epithelial–mesenchymal transition (EMT) is a central tumor-intrinsic pathway that drives invasiveness, metastasis, and therapy resistance in PTC (21). Key EMT regulators, including *TGFB1*, *SNAI1*/*2*, *TWIST1*/*2*, and *ZEB1*/*2*, are frequently activated and associated with stromal remodeling and immune suppression (22, 23). In the current study, we described *PHTF2* and *SNAI1* as critical modulators of EMT in thyroid cancer cells, where their inhibition reduces proliferation, colony formation, and EMT-promoting gene expression while upregulating epithelial markers such as *CDH1* (24, 25). Considering the intricate interactions between cancer cells, the tumor microenvironment, and EMT programs, a comprehensive single-cell resolution analysis is needed to dissect cellular heterogeneity and molecular mechanisms underlying age-specific differences in PTC. Here, we performed scRNA-seq analysis of thyroid tumors from underage and adult patients to profile immune, epithelial, and stromal populations; examine EMT activation; and investigate the roles of *PHTF2* and *SNAI1* in regulating tumor cell behavior and TME remodeling. These analyses aim to provide mechanistic insights into PTC progression, immune evasion, and potential therapeutic vulnerabilities.

## Materials and methods

### Cell culture

The human thyroid cancer cell lines, TPC-1 and KTC-1, obtained from the Cell Bank of the Chinese Academy of Sciences (Shanghai, China), were grown in Dulbecco’s modified Eagle medium (Gibco, USA) containing 10% fetal bovine serum (FBS; Gibco) and 1% penicillin–streptomycin (Gibco). Cell incubation was carried out at 37°C in a humidified environment with 5% CO_2_.

### Cell proliferation assay

The Cell Counting Kit-8 (CCK-8) assay was employed to evaluate cell proliferation. Following transfection, NC, si-TPC-1, and si-KTC cells were plated into 96-well plates at 2 × 10^4^ cells per well. A subsequent 24-h incubation period allowed for cellular attachment prior to the assay.

### Colony formation assay

Following transfection, TPC-1 or KTC-1 cells were plated into six-well plates at low densities (1,500 or 500 cells per well), with the medium refreshed every 3–4 days. After a 14-day incubation period, the resulting colonies were fixed using 4% paraformaldehyde (15 min) and subsequently stained with 0.1% crystal violet (30 min). Only colonies containing more than 50 cells were manually enumerated. All experimental conditions were assessed in triplicate. Mycoplasma contamination was routinely checked every two weeks. The pLKO-shRNA plasmid was a gift from D Anastasiou (Addgene #42516, USA), while the scramble shRNA control was based on sequences from Mission RNAi (Sigma, USA). Murine primer sequences are listed in the supplemental material (see section on Supplementary materials given at the end of the article).

### Western blotting

Cells were lysed on ice using a buffer containing 25 mM Tris–HCl (pH 8.0), 150 mM NaCl, 1 mM CaCl_2_, and 1% Triton X-100, supplemented with protease inhibitors (1:100, Bimake, B14001, USA). Following centrifugation, the supernatant’s protein concentration was quantified. Equal amounts of protein were separated by SDS-PAGE and transferred onto PVDF membranes (Millipore, Germany). After blocking with 5% non-fat milk, the membranes were probed with specific primary antibodies, followed by incubation with appropriate HRP-conjugated secondary antibodies. Protein bands were detected using an ECL system (Proteintech, USA). The following antibodies were used for western blotting E-cadherin, N-cadherin, and actin.

### RNA isolation and quantitative PCR

Total RNA was isolated from 10 mg of tissue samples using TRIzol reagent (Invitrogen, USA). Following purification via ethanol precipitation, the RNA was reverse-transcribed into cDNA with the PrimeScriptTM RT Reagent Kit (Takara, RR047A, Japan). Quantitative PCR analyses were then carried out on an ABI-7900-384 system with TB Green Premix Ex TaqTM II (Takara, RR820A). The relative transcript levels were calculated by the ΔΔCt values, using *β*-actin for normalization, and are presented as fold changes compared with the control group. All primer sequences can be found in Supplementary Table 1.

### Collection of single-cell RNA sequencing and GEO datasets

This study integrated three publicly available single-cell RNA sequencing datasets (GSE281736, GSE241184, and GSE250521), comprising a total of 20 samples from 10 patients. The dataset includes 6 underage normal (UN) samples (GSE281736: UN1-UN5; GSE241184: UN6) and their paired underage tumor (UT) samples (GSE281736: UT1-UT5; GSE241184: UT6), alongside 4 adult normal (AN) samples (GSE281736: AN1; GSE250521: AN2-AN4) and their paired adult tumor (AT) samples (GSE281736: AT1; GSE250521: AT2-AT4). Clinical data, including age, gender, stage, overall survival (OS), and vital status for thyroid carcinoma (THCA) patients, were obtained from the UCSC Xena website (https://xenabrowser.net/datapages/).

### Single-cell analysis and cell clustering

An in-depth bioinformatic interrogation of the single-cell RNA sequencing data matrix was carried out with the Scanpy package (version 1.9.1). Principal component analysis (PCA) served as the initial dimensionality reduction technique, configured via ov.pp.pca with n_pcs = 50 to encapsulate major sources of transcriptional heterogeneity. To correct for batch effects across samples, Harmony integration was applied to the PCA embeddings, enabling alignment of cells from different patients while preserving biological variation. Subsequently, a cell–cell neighborhood graph was constructed based on the Harmony-corrected embeddings, with n_neighbors = 15 to define local cellular relationships. The Leiden graph-based clustering algorithm was then employed for community detection within this network, using a resolution parameter of 0.2 to obtain biologically meaningful partitions. Resulting cluster identities were projected into two dimensions for visualization through Uniform Manifold Approximation and Projection (UMAP) plots generated by sc.pl.umap. Finally, automated cell type annotation was performed by the CellTypist tool, which leverages a reference database of marker genes to assign biological identities to each cluster.

### Cell type annotation

Initial cell type annotation was performed using the CellTypist database (version 2.0). Immune cells were annotated with the ‘Immune_All_High.pkl’ and ‘Immune_All_Low.pkl’ models. Due to the lack of a comprehensive, thyroid-specific reference at the time of analysis, epithelial cells were annotated using the ‘Human_Lung_Atlas.pkl’ model as a reference. All automated annotations were subsequently subjected to expert manual curation. This curation process involved validation against the expression of canonical lineage marker genes, including EPCAM for epithelial cells, PECAM1 for endothelial cells, COL1A1 for fibroblasts, CD3D for T cells, and CD68 for macrophages. Any discrepancy between the model prediction and the expression pattern of canonical markers was resolved in favor of the marker gene evidence.

### Construction and validation of the prognostic signature

We performed univariate Cox regression to screen for marker genes significantly linked to OS in TCGA-THCA patients, establishing a preliminary prognostic model from invasion-related genes. A *P*-value cutoff of <0.05 was set for selecting significant genes. We then applied the least absolute shrinkage and selection operator (LASSO) Cox regression via the ‘glmnet’ R package to further refine the gene set and pinpoint those with the greatest prognostic impact. A definitive risk score was calculated by linearly combining the expression levels of the finalized genes with their respective LASSO-derived coefficients. This approach yielded nine key prognostic genes. Patients were dichotomized into high-ESS (EMT signature score) and low-ESS groups based on the median risk score. Finally, we evaluated the model’s predictive performance and clinical applicability by constructing receiver operating characteristic (ROC) curves.

### Survival analysis

Kaplan–Meier analysis confirmed a significant association between the high-risk group and poorer OS. To assess this association, we generated survival curves using the ‘survival’ and ‘survminer’ R packages, evaluating the prognostic relevance of the risk score-associated genes within the TCGA-THCA cohort.

### Gene ontology and KEGG pathway analysis

Gene ontology (GO) enrichment analysis was carried out with the clusterProfiler R package (v3.18.1) and the org.Hs.eg.db annotation database. Differentially expressed genes (DEGs), after conversion from gene symbols to Entrez IDs via the bitr function, were analyzed. The enrichGO function evaluated enrichment across three ontologies, biological process (BP), cellular component (CC), and molecular function (MF), using these parameters: OrgDb = org.Hs.eg.db, *P*valueCutoff = 0.05, qvalueCutoff = 0.05, readable = TRUE. Significantly enriched GO terms, determined by an adjusted *P*-value (Benjamini–Hochberg method) below 0.05, were visualized in dot and bar plots. Concurrently, KEGG pathway analysis was performed through the g:Profiler Python interface (gprofiler-official). Submitted gene lists underwent enrichment testing against the default human annotation (hsapiens). Statistically significant pathways (FDR < 0.05) were identified and graphically represented to illuminate relevant biology.

### Differentially expressed gene analysis

We performed differential gene expression analysis for every cell cluster against remaining cells using both the DESeq2 package and the Wilcoxon rank-sum test. Significance was determined by a *P*-value <0.05 and a log (fold change) ≥ 2 or ≤ −2 for categorizing genes as upregulated or downregulated.

### Gene set enrichment analysis (GSEA)

To identify significantly enriched pathways, we performed gene set enrichment analysis (GSEA) employing the hallmark gene sets (h.all.v2023.2.Hs.symbols.gmt) obtained from MSigDB (http://software.broadinstitute.org/gsea/msigdb/).

### Gene set variation analysis (GSVA)

GSVA was employed to compare the activity of enriched pathways between the high-ESS and low-ESS groups. Using the ‘fgsea’ R package, single-sample GSEA (ssGSEA) scores for each gene set were calculated across all TCGA-BRCA samples.

### Calculation of EMT prognostic signature

The EMT signature score for each tumor sample was calculated using the ssGSEA algorithm implemented in the GSVA R package, with the gene set ‘HALLMARK_EPITHELIAL_MESENCHYMAL_TRANSITION’ from the Molecular Signatures Database (MSigDB). This process was applied to the bulk RNA-seq data from the TCGA-THCA cohort. To categorize samples, the median ssGSEA score across all tumors was used as the threshold, defining ‘EMT-high’ (score > median) and ‘EMT-low’ (score ≤ median) groups. This stratification was subsequently used for all differential expression and correlation analyses.

### Integrated analysis of the TME and therapeutic vulnerabilities

We applied the ESTIMATE and TIDE algorithms to evaluate immune/stromal infiltration, tumor purity, and potential immune evasion within the tumor microenvironment. T cell receptor (TCR) repertoire analysis was conducted to assess T cell diversity, specificity, and reactivity, with diversity and richness quantified as previously described. Furthermore, immune cell subtype abundances were estimated using TIMER2.0 and CIBERSORT, revealing detailed infiltration patterns. Drug sensitivity was predicted with the pRRophetic package, and the drug data 2016 resource enabled tissue-specific drug filtering. Together, these multifaceted approaches provided a comprehensive perspective on the tumor microenvironment and its therapeutic implications.

### Statistical analysis

Statistical comparisons were performed with a two-tailed Student’s t-test implemented in R. Multivariate analysis was conducted using a Cox proportional hazards regression model via the ‘survival’, ‘survminer’, and ‘forestplot’ R packages to identify independent prognostic factors for OS in the TCGA-THCA cohort. The false discovery rate (FDR) method was applied for multiple testing correction, with an FDR or raw *P*-value below 0.05 considered statistically significant. An adjusted *P*-value < 0.05 was used as the significance threshold. Data are expressed as mean ± SEM. For experiments repeated in at least three independent replicates, an unpaired two-tailed Student’s *t*-test was used for significance testing in GraphPad Prism (GraphPad Software, USA). A *P*-value less than 0.05 was regarded as statistically significant. *P < 0.05; **P < 0.01; ***P < 0.001.

## Results

### Cellular atlas of thyroid cancer across age groups

**Figure 1:**
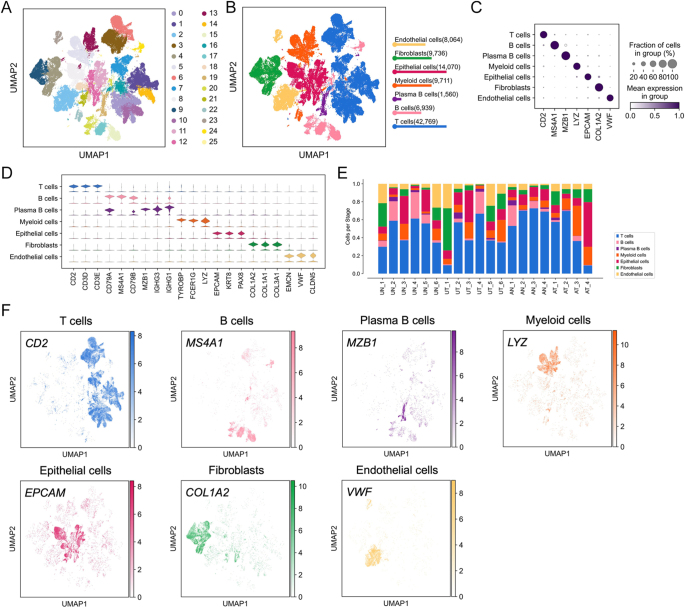
Single-cell transcriptomic landscape of thyroid cancer. (A) UMAP visualization of 25 clusters. (B) Annotation of seven major cell types using canonical markers. (C) Dot plot of representative markers across cell types. (D) Violin plots showing expression of selected markers. (E) Stacked bar plots illustrating cell type proportions across samples. (F) Feature plots of canonical marker genes validating cell type annotation. A full color version of this figure is available at https://doi.org/10.1530/ERC-26-0021.

Single-cell transcriptomic profiling of thyroid cancer tissues, comprising 92,849 cells, identified 26 distinct clusters on the UMAP visualization (Fig. 1A). Based on canonical marker expression, these clusters were annotated into seven major cell types, including T cells (*CD2*), B cells (*MS4A1*), plasma B cells (*MZB1*), myeloid cells (*LYZ*), epithelial cells (*EPCAM*), fibroblasts (*COL1A2*), and endothelial cells (*VWF*) (Fig. 1B and C). The specificity of marker expression confirmed the accuracy of classification (Fig. 1D). Analysis of cellular composition revealed pronounced inter-patient heterogeneity in cell type proportions (Fig. 1E), indicating variability in immune infiltration, tumor progression, and therapeutic response. After cross-sample integration of 20 thyroid tissue samples, including 6 UN (underage normal), 6 UT (underage tumor), 4 AN (adult normal), and 4 AT (adult tumor), cells were evenly distributed across individuals (Fig. S1A and B), supporting robust integration quality. Correlation analysis (Fig. S1C) demonstrated that immune cells (T, B, and plasma B cells) were more closely related to each other, whereas non-immune cells (epithelial cells, fibroblasts, and endothelial cells) formed another distinct group. A single-cell heatmap (Fig. S1D) further validated marker specificity across cell populations. Collectively, these findings highlight that the thyroid cancer microenvironment comprises both immune and non-immune compartments, with inter-patient variability reflecting tumor heterogeneity and immune evasion. Altered transcriptional states of epithelial cells may drive tumorigenesis, while fibroblasts and endothelial cells underscore the roles of stromal remodeling and angiogenesis in thyroid cancer progression.

### T cell heterogeneity and immunoregulatory reprogramming

**Figure 2:**
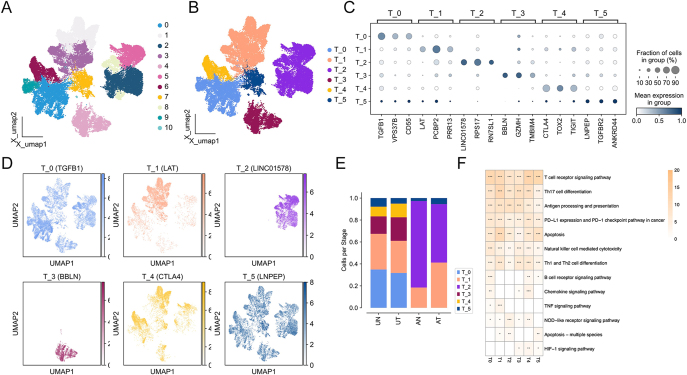
Identification and functional characterization of T cell subsets in thyroid cancer. (A) UMAP plot showing 11 T cell clusters. (B) Classification of clusters into six subsets (T_0–T_5). (C) Dot plot displaying representative marker genes of each subset. (D) UMAP plots of subtype-specific marker gene expression. (E) Proportion of T cell subsets across UN, UT, AN, and AT groups. (F) Pathway enrichment analysis of T cell subsets highlighting immune-related signaling pathways. A full color version of this figure is available at https://doi.org/10.1530/ERC-26-0021.

Analysis of T cells from thyroid cancer single-cell transcriptomes identified 11 transcriptionally distinct clusters, which were consolidated into six major subsets (T_0–T_5) defined by representative markers, including *TGFB1*, *LAT*, *LINC01578*, *BBLN*, *CTLA4*, and *LNPEP* (Fig. 2A, B, C, D). Cross-sample integration confirmed the robustness of T cell subtype representation across UN, UT, AN, and AT groups (Fig. S2A), and heatmap analysis highlighted distinct marker expression signatures that distinguished each subset (Fig. S2B). Distribution profiling demonstrated stage-specific enrichment patterns, with T_0 and T_1 subsets predominating in underage samples, whereas T_2 and T_5 subsets were enriched in adult tumors (Figs 2E and S2C). Functional annotation revealed that these subsets were engaged in diverse immune processes, including T cell receptor signaling, Th17 differentiation, PD-1/PD-L1 checkpoint regulation, and apoptosis pathways (Fig. 2F). GO enrichment further underscored the immunoregulatory features of TGFB1+ (T_0) and CTLA4+ (T_4) subsets, implicating them in negative regulation of T cell activation, tolerance induction, and cytokine modulation (Fig. S2D). Collectively, these findings delineate the coexistence of effector and immunosuppressive T cell programs in the thyroid cancer microenvironment, suggesting that functional reprogramming of T cells contributes to immune evasion and may underlie the clinical divergence between underage and adult patients.

### Epithelial cell heterogeneity in thyroid cancer

**Figure 3:**
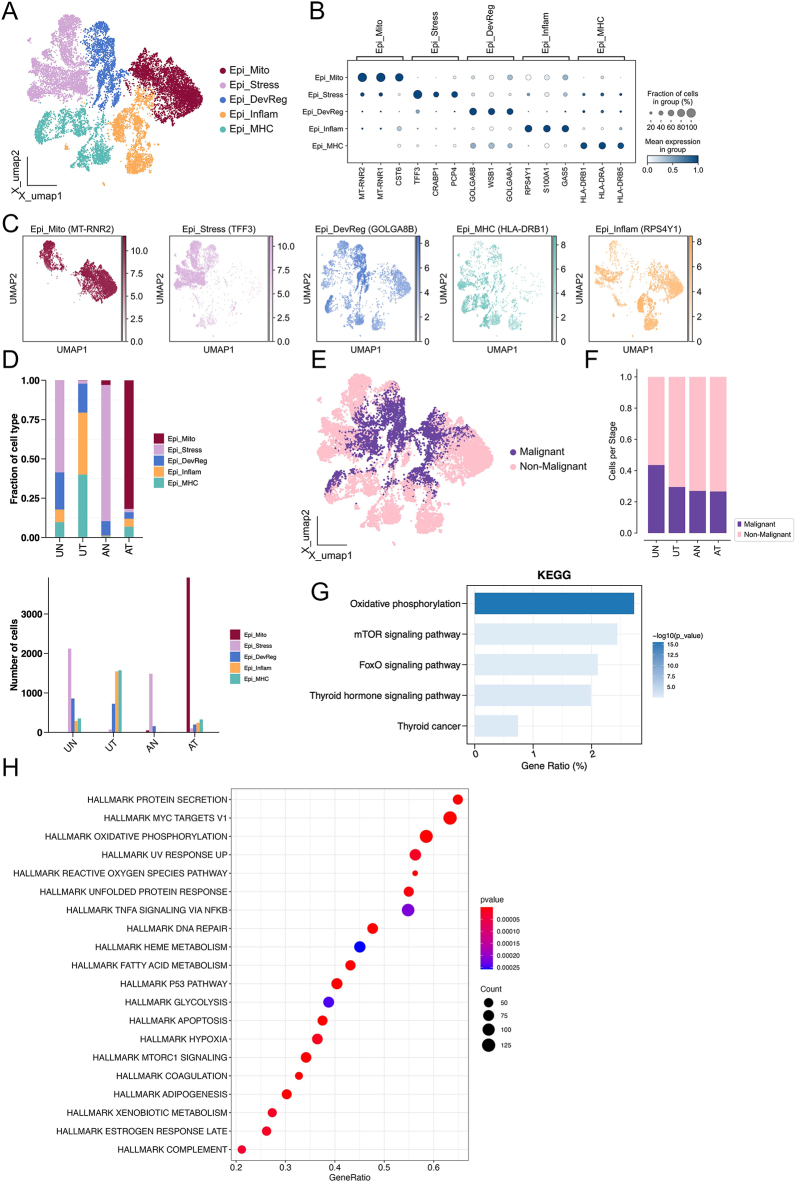
Functional heterogeneity of thyroid cancer epithelial cells. (A) Epithelial cell heterogeneity in thyroid cancer. (A) UMAP plot showing five epithelial cell subsets (Epi_Mito, Epi_Stress, Epi_DevReg, Epi_Inflam, and Epi_MHC). (B and C) Dot plots and feature plots displaying representative markers for each subset. (D) Distribution of epithelial subsets across UN, UT, AN, and AT samples. (E and F) UMAP and bar plots showing malignant and non-malignant epithelial cells. (G) KEGG enrichment analysis of epithelial subsets. (H) Hallmark pathway enrichment highlighting functional heterogeneity. A full color version of this figure is available at https://doi.org/10.1530/ERC-26-0021.

Subclustering analysis of epithelial cells identified five transcriptionally distinct subsets, annotated as Epi_Mito, Epi_Stress, Epi_DevReg, Epi_Inflam, and Epi_MHC, defined by representative markers such as *MT-RNR2*, *TFF3*, *GOLGA8B*, *RPS4Y1*, and *HLA-DRB1* (Fig. 3A, B, C). Heatmap and dot plot analyses further confirmed discrete gene expression patterns distinguishing these subtypes (Fig. S3D and E). Distribution profiling revealed stage-specific enrichment, with Epi_Mito and Epi_Stress subsets expanded in adult tumors, whereas Epi_DevReg and Epi_MHC subsets were more abundant in underage samples (Figs 3D and S3C). Malignant epithelial cells were predominantly distributed in adult tumor tissues (Fig. 3E and F). Functional enrichment analyses indicated that Epi_Mito cells were strongly associated with oxidative phosphorylation and metabolic pathways, while Epi_Stress cells showed activation of protein processing and stress-response pathways (Figs 3G, H and S3F). In addition, Epi_DevReg and Epi_MHC subsets were enriched for immune-modulatory functions, including antigen presentation and regulation of ER-related processes, whereas Epi_Inflam cells exhibited transcriptional signatures of pro-inflammatory cytokine signaling. These findings highlight pronounced epithelial cell heterogeneity in thyroid cancer, suggesting that distinct epithelial programs contribute to tumor progression through metabolic reprogramming, stress adaptation, immune evasion, and inflammatory activation, ultimately influencing differences between underage and adult patients.

### Tumor-associated macrophage heterogeneity and immune modulation

**Figure 4:**
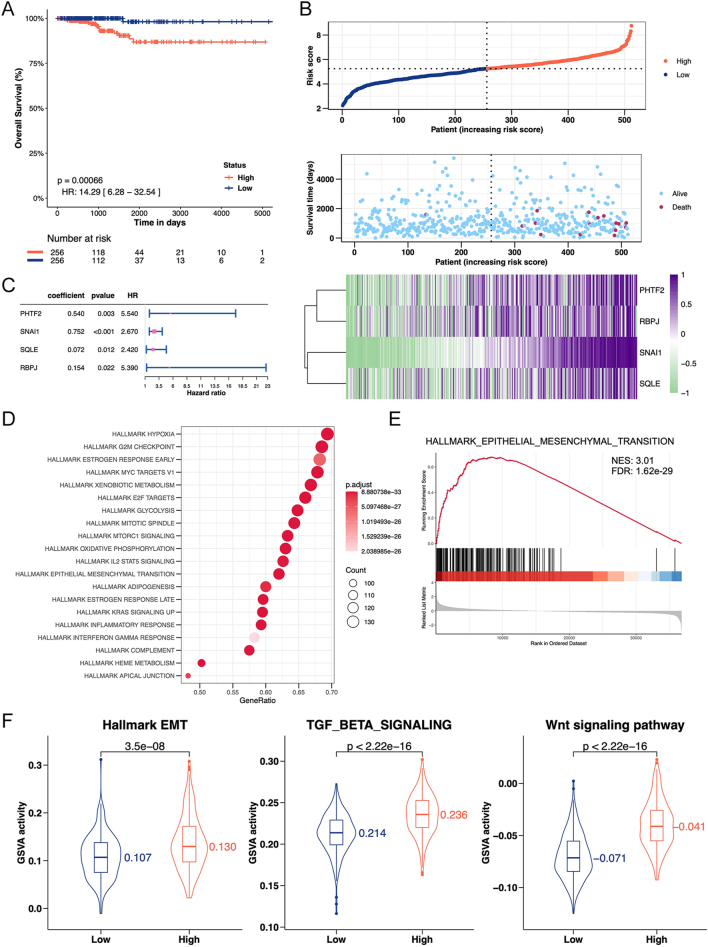
Transcriptional heterogeneity of TAMs in thyroid cancer. (A) UMAP visualization of macrophage subsets identified by clustering analysis. (B) Marker gene expression patterns distinguishing each TAM subset. (C) Heatmap of representative subset-specific markers. (D) Distribution of TAM subsets across underage (U) and adult (A) samples. (E) Proportional differences of TAM subsets in normal (N) versus tumor (T) groups. (F) GO and KEGG enrichment analyses highlighting functional pathways of selected TAM subsets. A full color version of this figure is available at https://doi.org/10.1530/ERC-26-0021.

Macrophage profiling revealed marked heterogeneity within tumor-associated macrophages (TAMs), which segregated into subsets with transcriptional programs reflective of inflammatory, immunosuppressive, and antigen-presenting states (Fig. 4A, B, C). Comparative distribution analyses demonstrated enrichment of pro-inflammatory TAM subsets in underage tumors, whereas immunoregulatory TAM clusters predominated in adult thyroid cancer (Fig. 4D and E), indicating age-specific macrophage polarization. Functional annotation highlighted key involvement of antigen processing and presentation, NF-κB signaling, and immune checkpoint pathways (Fig. 4F). Supplementary validation confirmed subtype reproducibility across samples (Fig. S4A) and revealed distinct marker gene expression patterns (Fig. S4B). These findings delineate the dual roles of TAMs in orchestrating both anti-tumor and immunosuppressive programs, suggesting that TAM heterogeneity contributes to shaping the tumor immune microenvironment and may underlie differential therapeutic responses between underage and adult patients.

### Tumor microenvironment remodeling and EMT activation

**Figure 5:**
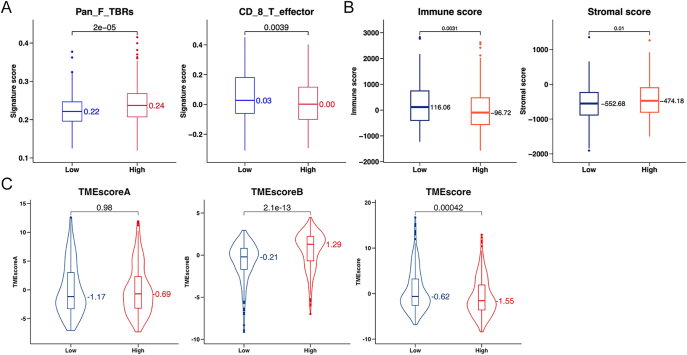
Tumor microenvironment remodeling and EMT activation in high-signature tumors. (A) Comparison of fibroblast-associated Pan_F_TBRs scores and CD8+ T effector activity between low- and high-signature groups. (B) Immune and stromal scores across subgroups. (C) TMEscoreA, TMEscoreB, and composite TMEscore profiling. A full color version of this figure is available at https://doi.org/10.1530/ERC-26-0021.

Transcriptomic stratification based on epithelial signatures revealed profound alterations in the tumor microenvironment (TME). High-signature tumors displayed significantly elevated fibroblast-associated Pan_F_TBRs scores and stromal components, whereas CD8+ T effector activity was markedly reduced, suggesting an immune-excluded phenotype (Fig. 5A). Consistently, immune and stromal scores were higher in the high group, accompanied by increased TMEscoreB and overall TMEscore, indicating extensive TME remodeling toward a stroma-rich, immunosuppressive state (Fig. 5B and C). To further investigate the molecular underpinnings of this phenotype, we evaluated epithelial–mesenchymal transition (EMT)-related regulators. High-signature tumors showed transcriptional activation of canonical EMT drivers, including *TGFB1*, *SNAI1*, *SNAI2* (*SLUG*), *TWIST1/2*, and *ZEB1/2*, reflecting a phenotypic shift toward mesenchymal states (Fig. S5A and B). Notably, these EMT alterations were not associated with changes in tumor purity, suggesting that EMT activation was intrinsic to tumor epithelial cells rather than a reflection of altered tumor content (Fig. S5C). Collectively, these findings highlight that epithelial-derived programs are tightly coupled with both TME remodeling and EMT activation, which may synergistically promote tumor invasiveness and immune evasion in thyroid cancer.

### Drug sensitivity prediction in EMT-high tumors

**Figure 6:**
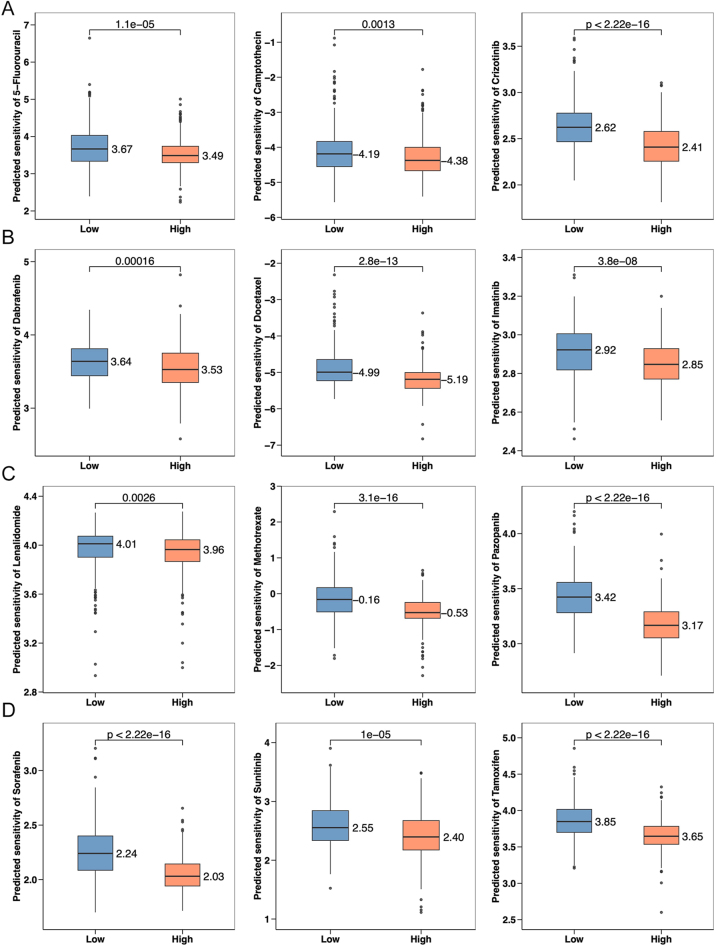
Predicted drug sensitivity analysis between high-EMT and low-EMT groups. (A) Sensitivity comparison of 5-fluorouracil, camptothecin, and crizotinib between high-EMT and low-EMT. (B) Sensitivity differences to dabrafenib, docetaxel, and imatinib between high-EMT and low-EMT. (C) Predicted sensitivity to lenalidomide, methotrexate, and pazopanib across high-EMT and low-EMT. (D) Predicted drug sensitivity to sorafenib, sunitinib, and tamoxifen across high-EMT and low-EMT. A full color version of this figure is available at https://doi.org/10.1530/ERC-26-0021.

Drug sensitivity prediction revealed potential therapeutic implications in thyroid cancer. Patients with high EMT signatures exhibited reduced sensitivity to multiple conventional chemotherapeutics, including 5-fluorouracil, docetaxel, and methotrexate, as well as targeted therapies such as crizotinib, imatinib, sorafenib, and sunitinib (Fig. 6A and B). In contrast, sensitivity to camptothecin and dabrafenib was significantly enhanced in the high-EMT group (Fig. 6C and D). These findings suggest that EMT-associated transcriptional programs may contribute not only to invasive and immune-evasive tumor phenotypes but also to altered therapeutic responses, underscoring the potential value of incorporating EMT status into treatment stratification. It should be noted, however, that these drug sensitivity analyses are based on *in silico* predictions derived from transcriptional signatures and therefore may not directly translate into clinical response; accordingly, these results should be interpreted with caution. Targeting EMT-associated vulnerabilities may nevertheless provide promising avenues for therapeutic intervention in refractory thyroid cancer.

### *PHTF2* and *SNAI1* regulate EMT pathway activity

**Figure 7:**
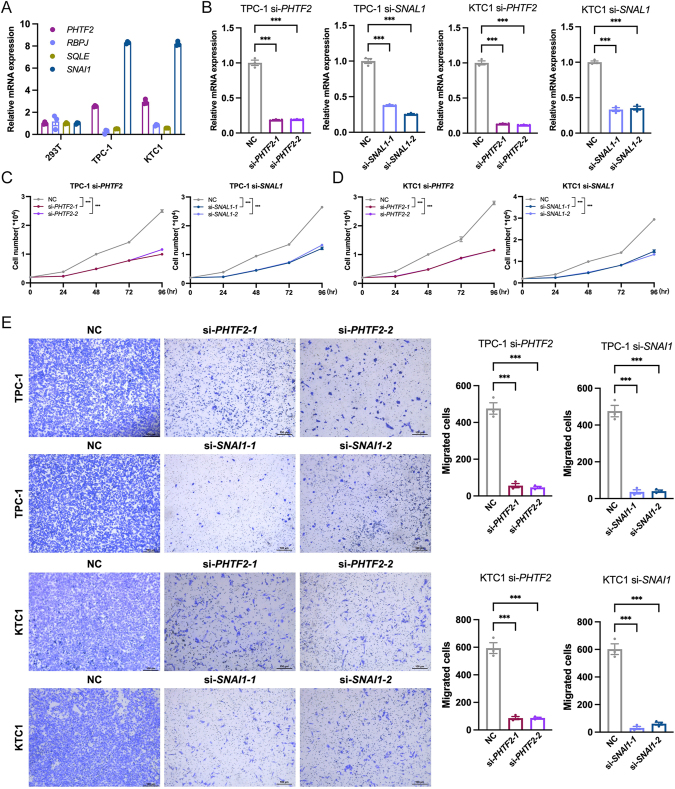
*PHTF2* and *SNAI1* promote proliferation and migration of thyroid cancer cells. (A) Relative mRNA expression of *PHTF2* and *SNAI1* in thyroid cancer cell lines (TPC-1 and KTC-1) compared with 293T cells. (B) Knockdown efficiency of three independent siRNAs targeting *PHTF2* and *SNAI1* confirmed by qRT-PCR in TPC-1 and KTC-1 cells. (C and D) Cell proliferation assays showing reduced growth rates following *PHTF2* or *SNAI1* silencing in TPC-1 (C) and KTC-1 (D) cells. (E) Transwell migration assays demonstrating significantly decreased migratory capacity after *PHTF2* or *SNAI1* knockdown. Data are presented as mean ± SD from three independent experiments. *P* < 0.05, *P* < 0.01, *P* < 0.001 vs NC group. A full color version of this figure is available at https://doi.org/10.1530/ERC-26-0021.

To elucidate the functional significance of *PHTF2* and *SNAI1* in thyroid cancer, we first examined their expression in multiple thyroid cancer cell lines. Both genes were markedly upregulated in TPC-1 and KTC-1 cells compared with 293T cells (Fig. 7A). To determine their biological roles, three independent siRNAs were designed for each gene, and efficient knockdown was confirmed by qRT-PCR in both cell lines (Fig. 7B). Functional assays revealed that silencing either *PHTF2* or *SNAI1* significantly impaired thyroid cancer cell proliferation. Growth curve analysis demonstrated a time-dependent decrease in viable cell numbers in both TPC-1 and KTC-1 cells following knockdown, with significant suppression evident by 72–96 h (Fig. 7C and D). Furthermore, migration assays showed that depletion of *PHTF2* or *SNAI1* markedly reduced the number of migrated cells (Fig. 7E), indicating that both genes are essential for maintaining the proliferative and migratory capacities of thyroid cancer cells.

**Figure 8:**
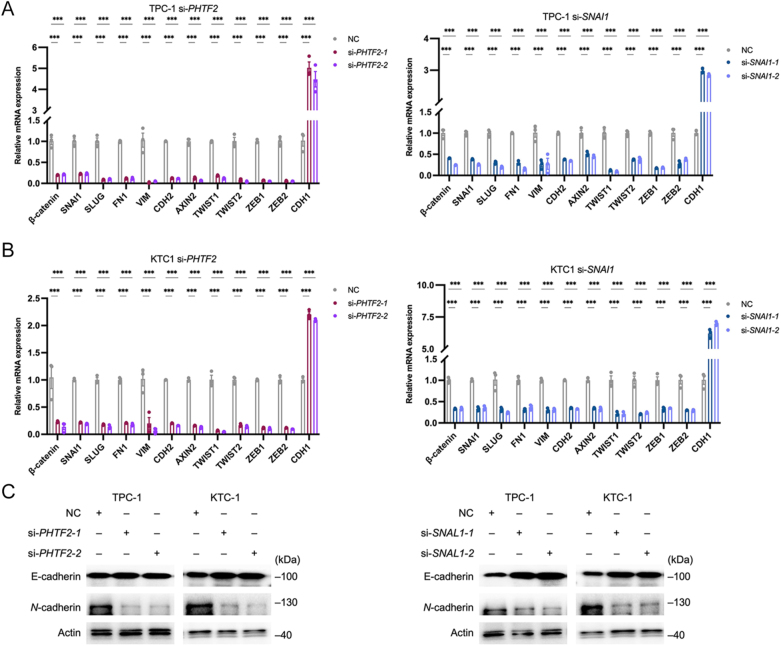
Knockdown of *PHTF2* or *SNAI1* suppresses EMT-related gene expression in thyroid cancer cells. (A and B) qRT-PCR analysis showing downregulation of mesenchymal and EMT-promoting genes (*β*-catenin, *SNAI1*, *SLUG*, *FN1*, *VIM*, *CDH2*, *AXIN2*, *TWIST1*, *TWIST2*, *ZEB1*, and *ZEB2*) and upregulation of epithelial marker *CDH1* following *PHTF2* or *SNAI1* knockdown in TPC-1 (A) and KTC-1 (B) cells. (C) Western blot analysis confirming increased E-cadherin and decreased N-cadherin protein levels upon *PHTF2* (left) or *SNAI1* (right) silencing. Data are shown as mean ± SD from three independent experiments. *P* < 0.05, *P* < 0.01, *P* < 0.001. A full color version of this figure is available at https://doi.org/10.1530/ERC-26-0021.

To further explore the molecular mechanisms underlying these phenotypic effects, we assessed the expression of EMT markers following *PHTF2* or *SNAI1* silencing. qRT-PCR analyses demonstrated consistent transcriptional reprogramming in both TPC-1 and KTC-1 cells. Knockdown of either gene significantly downregulated mesenchymal and EMT-promoting genes, including β-catenin, *SNAI1*, *SLUG* (*SNAI2*), *FN1* (fibronectin-1), *VIM* (vimentin), *CDH2* (*N*-cadherin), *AXIN2*, *TWIST1*, *TWIST2*, *ZEB1*, and *ZEB2*, while concurrently upregulating the epithelial marker *CDH1* (E-cadherin) (Fig. 8A and B). Western blot analysis further confirmed these results, showing increased E-cadherin and decreased *N*-cadherin protein levels upon *PHTF2* or *SNAI1* knockdown (Fig. 8C). Collectively, these data demonstrate that *PHTF2* and *SNAI1* cooperatively promote EMT activation and tumor aggressiveness in thyroid cancer. Their silencing restores epithelial characteristics, suppresses proliferation and migration, and reverses mesenchymal transition, underscoring their potential as therapeutic targets for EMT-driven thyroid cancer progression.

## Discussion

Thyroid cancer, with PTC as its most common subtype, demonstrates significant cellular and molecular heterogeneity, which contributes to its diverse clinical behavior. Although PTC typically carries an excellent prognosis, disease recurrence and distant metastasis pose substantial clinical management difficulties. This underscores the importance of elucidating the interplay between tumor cell-intrinsic pathways and the TME in driving cancer aggressiveness (26, 27). In the present investigation, we performed scRNA-seq analysis on thyroid tumor specimens obtained from both pediatric/young adult and adult patients. This approach identified 26 discrete cell clusters, representing immune, stromal, and epithelial lineages. Our results uncovered pronounced age-associated disparities in tumor composition, transcriptional profiles, and functional pathway activities, yielding novel perspectives on the mechanisms governing tumor progression, immune evasion, and treatment resistance in PTC.

Importantly, to better position our study relative to existing literature, we explicitly define its conceptual advances in three aspects. First, our study provides a systematic age-stratified comparison between pediatric/young and adult PTC at single-cell resolution, an area that remains relatively underexplored. Second, we integrate EMT-associated transcriptional programs with immune and stromal remodeling within the tumor microenvironment in an age-dependent context, rather than examining these components in isolation. Third, we identify PHTF2 as a previously underappreciated factor associated with EMT-related transcriptional programs in thyroid cancer. Collectively, these elements distinguish our work from prior studies and provide a more integrative framework for understanding tumor progression.

Our data reveal significant heterogeneity across both tumor cells and the surrounding TME. T cell characterization delineated six principal subsets defined by unique transcriptional signatures associated with either effector functions or immunosuppression. Specifically, the TGFB1^+^ (T_0) and CTLA4^+^ (T_4) subsets displayed robust immunoregulatory properties, suggesting their role in actively suppressing antitumor immune responses within the tumor microenvironment. The distribution of these subsets exhibited age-dependent variation: tumors in underage patients showed enrichment of effector T cell programs, whereas adult tumors were dominated by immunosuppressive T cell populations. Similarly, TAM demonstrated a polarization shift toward pro-inflammatory states in younger patients and immunoregulatory states in adults (28). These findings corroborate existing evidence of age-dependent immune alterations in papillary thyroid carcinoma and highlight how the composition of the tumor microenvironment can influence disease progression and potentially shape responses to immunotherapy.

Subclustering of epithelial cells revealed five transcriptionally distinct subsets (Epi_Mito, Epi_Stress, Epi_DevReg, Epi_Inflam, and Epi_MHC), each encoding unique functional programs. The Epi_Mito and Epi_Stress subsets, which were notably enriched in adult tumors, displayed metabolic and stress-response signatures suggestive of adaptive processes that could promote tumor progression. Conversely, underage tumors harbored greater proportions of Epi_DevReg and Epi_MHC cells, which were implicated in antigen presentation and ER-related regulatory functions, suggesting heightened immunogenicity and distinct epithelial plasticity. Thus, the integration of metabolic reprogramming, stress adaptation, and immune modulation underpins epithelial heterogeneity and provides a mechanistic basis for age-dependent differences in PTC aggressiveness (29, 30).

Among tumor-intrinsic signaling pathways, EMT emerged as a pivotal mechanism underlying tumor invasiveness, immune evasion, and treatment resistance (31). Tumors with high EMT activity displayed stromal enrichment, diminished CD8^+^ T cell effector function, and upregulated expression of key EMT regulators such as *TGFB1*, *SNAI1*/*2*, *TWIST1*/*2*, and *ZEB1*/*2*. Functional studies further established *PHTF2* and *SNAI1* as critical modulators of EMT in thyroid cancer cells. Knockdown of *PHTF2* or *SNAI1* markedly impaired cellular proliferation and clonogenicity, while upregulating epithelial markers such as *CDH1* and downregulating key EMT-promoting genes (including β-catenin, *SNAI2*/*SLUG*, *FN1*, *VIM*, *CDH2*, *AXIN2*, *TWIST1*/*2*, and *ZEB1*/*2*). These findings position *PHTF2* and *SNAI1* as upstream regulators orchestrating EMT-driven transcriptional reprogramming and tumor microenvironment remodeling (32, 33). Given the established role of EMT in fostering stromal activation, immune suppression, and therapy resistance, therapeutic targeting of *PHTF2* or *SNAI1* may represent a promising approach to disrupt these pro-tumorigenic networks and improve treatment outcomes.

The interaction among EMT, stromal remodeling, and immune modulation represents a key finding. Tumors with elevated EMT activity showed enriched fibroblast-associated signatures and higher stromal scores, alongside suppressed CD8^+^ T cell effector function, supporting a role for EMT in fostering an immune-excluded tumor microenvironment (34, 35). This finding is consistent with previous studies demonstrating that mesenchymal cancer cells recruit immunosuppressive stromal components and impede cytotoxic T cell recruitment, thereby promoting disease progression and metastatic dissemination. Furthermore, a high EMT status predicted reduced susceptibility to various drugs, including 5-fluorouracil, docetaxel, methotrexate, crizotinib, imatinib, sorafenib, and sunitinib, alongside increased sensitivity to camptothecin and dabrafenib (36, 37). These results affirm the clinical significance of EMT-mediated transcriptional reprogramming in determining treatment outcomes and point to EMT-linked vulnerabilities as promising avenues for therapeutic intervention in refractory PTC. It should be clarified that the EMT signature score in this study was not a directly measured single-cell feature but a quantification of the activity of this molecular program derived from bulk RNA-seq data. Our scRNA-seq analysis provides mechanistic context for why tumors stratified by this score might exhibit differential treatment responses. We hypothesize that the immunosuppressive and stromal-rich tumor microenvironment associated with EMT-high tumors (as evidenced by our scRNA-seq data) could contribute to chemoresistance by limiting drug penetration, fostering survival niches for cancer cells, or directly suppressing cytotoxic immune responses.

*PHTF2* and *SNAI1* may serve as critical nodes linking intrinsic EMT programs with TME remodeling. By regulating canonical EMT transcription factors, these genes likely influence stromal activation, extracellular matrix deposition, and immune suppression, creating a feedback loop that reinforces tumor aggressiveness. The consistent effects observed across TPC-1 and KTC-1 thyroid cancer cell lines support the robustness of these regulatory interactions and suggest that interventions targeting *PHTF2* or *SNAI1* could simultaneously inhibit tumor cell proliferation and mitigate immunosuppressive microenvironmental changes.

Despite these advances, several limitations merit consideration. First, our study relies primarily on scRNA-seq data, which, while providing high-resolution transcriptional profiles, cannot fully capture protein-level regulation, post-translational modifications, or spatially resolved cell–cell interactions. Although integration with spatial transcriptomics partially addresses spatial context, current platforms lack true single-cell resolution, limiting precise mapping of cellular crosstalk (38, 39, 40). Second, the functional validation of *PHTF2* and *SNAI1* was conducted *in vitro* using two thyroid cancer cell lines; future studies employing patient-derived xenograft models or organoid systems are necessary to confirm their physiological relevance *in vivo* (41, 42). Finally, our analysis focused on transcriptional programs and did not fully incorporate other regulatory layers, such as epigenetic modulation, metabolic rewiring, or non-coding RNA activity, that may also contribute to EMT, TME remodeling, and therapy resistance.

In summary, our study provides a comprehensive single-cell atlas of thyroid cancer across age groups, revealing marked heterogeneity in immune, epithelial, and stromal compartments. EMT emerges as a key driver of tumor aggressiveness, TME remodeling, and therapy resistance, with *PHTF2* and *SNAI1* identified as critical regulators of this process. These findings underscore the importance of integrating tumor-intrinsic and microenvironmental analyses to understand PTC progression, immune evasion, and therapeutic vulnerabilities. Targeting EMT-associated pathways, particularly through *PHTF2* and *SNAI1*, may offer promising avenues for precision therapy in PTC patients, especially those with aggressive or therapy-resistant disease. Future studies integrating multi-omic, spatial, and functional approaches are needed to fully elucidate the complex regulatory networks governing thyroid cancer progression and advance these findings toward clinical translation.

## Supplementary materials





## Declaration of interest

The authors declare that there is no conflict of interest that could be perceived as prejudicing the impartiality of the work reported.

## Funding

This work was supported by the Medical and Health Project of Zhejiang (2025KY1963) and Traditional Chinese Medicine Project of Zhejiang (2025ZL618).

## Author contribution statement

Qi Zhou performed the study concept and design; Qiankun Zhang developed the methodology; Qiankun Zhang, WP, and XG performed acquisition, analysis, and interpretation of data and statistical analysis; Qi Zhou and Qiankun Zhang wrote the manuscript. All authors performed writing, review, and revision of the paper and read and approved the final paper.

## Data availability

The single-cell RNA-seq dataset was sourced from the GEO database using accession numbers GSE241184, GSE250521, and GSE281736. We obtained the TCGA-THCA cohort dataset from The Cancer Genome Atlas (TCGA) portal (https://portal.gdc.cancer.gov/).
