# Supplementary material for: Single-cell reveals age-dependent epithelial reprogramming and EMT vulnerability in THCA
Source: Endocr Relat Cancer. 2026 Jun 8;33(6):e260021. doi: 10.1530/ERC-26-0021 (PMC13250642; doi:10.1530/ERC-26-0021)

**Supplementary Figure 1.** Validation of cell type identification and sample integration.

(A-B) UMAP plots showing cell distribution across individual samples and conditions. (C) Correlation heatmap of major cell types. (D) Heatmap of representative markers across all cells confirming cell type classification.

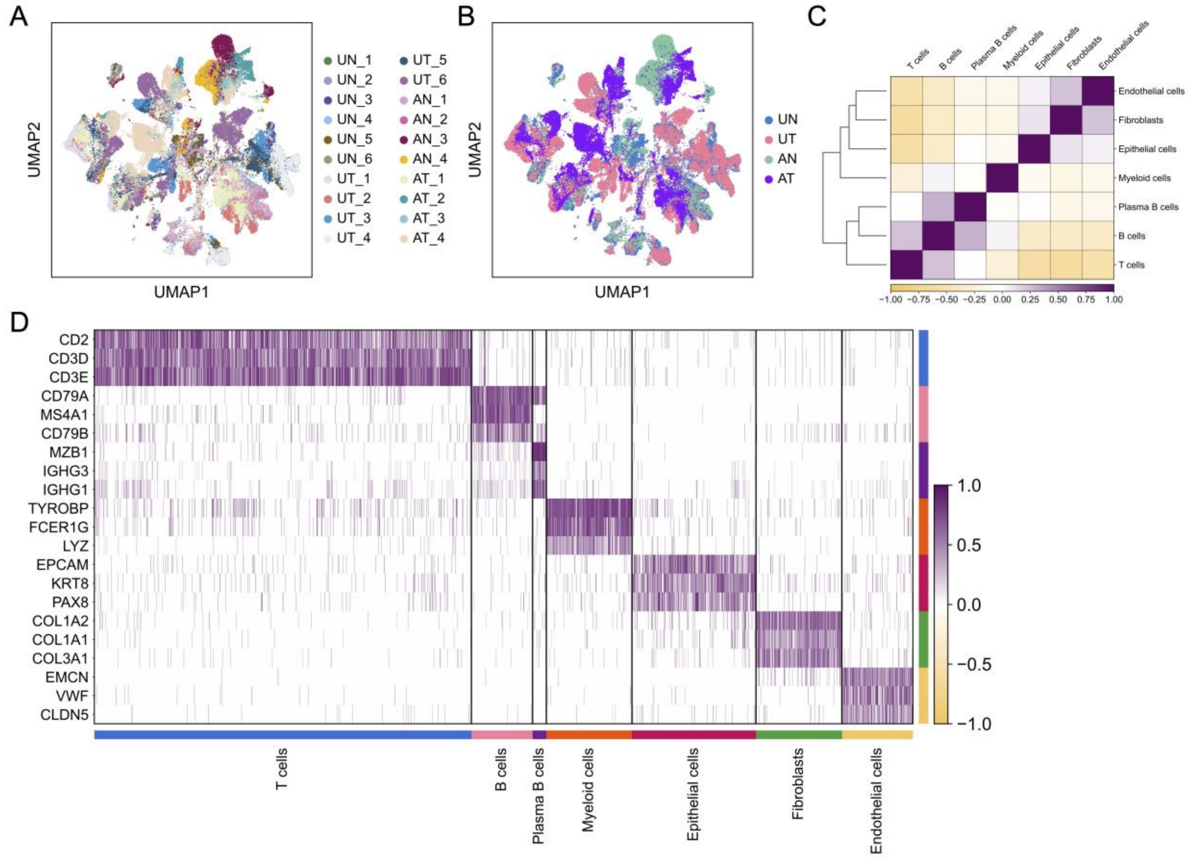

**Supplementary Figure 2.** Validation and functional annotation of T cell subsets. (A) UMAP projection showing T cell distribution across UN, UT, AN, and AT samples. (B) Heatmap of marker gene expression across T cell subsets. (C) Distribution of T cell subsets in individual samples. (D) GO enrichment analysis showing immunoregulatory processes including negative regulation of T cell activation, tolerance induction, and cytokine modulation.

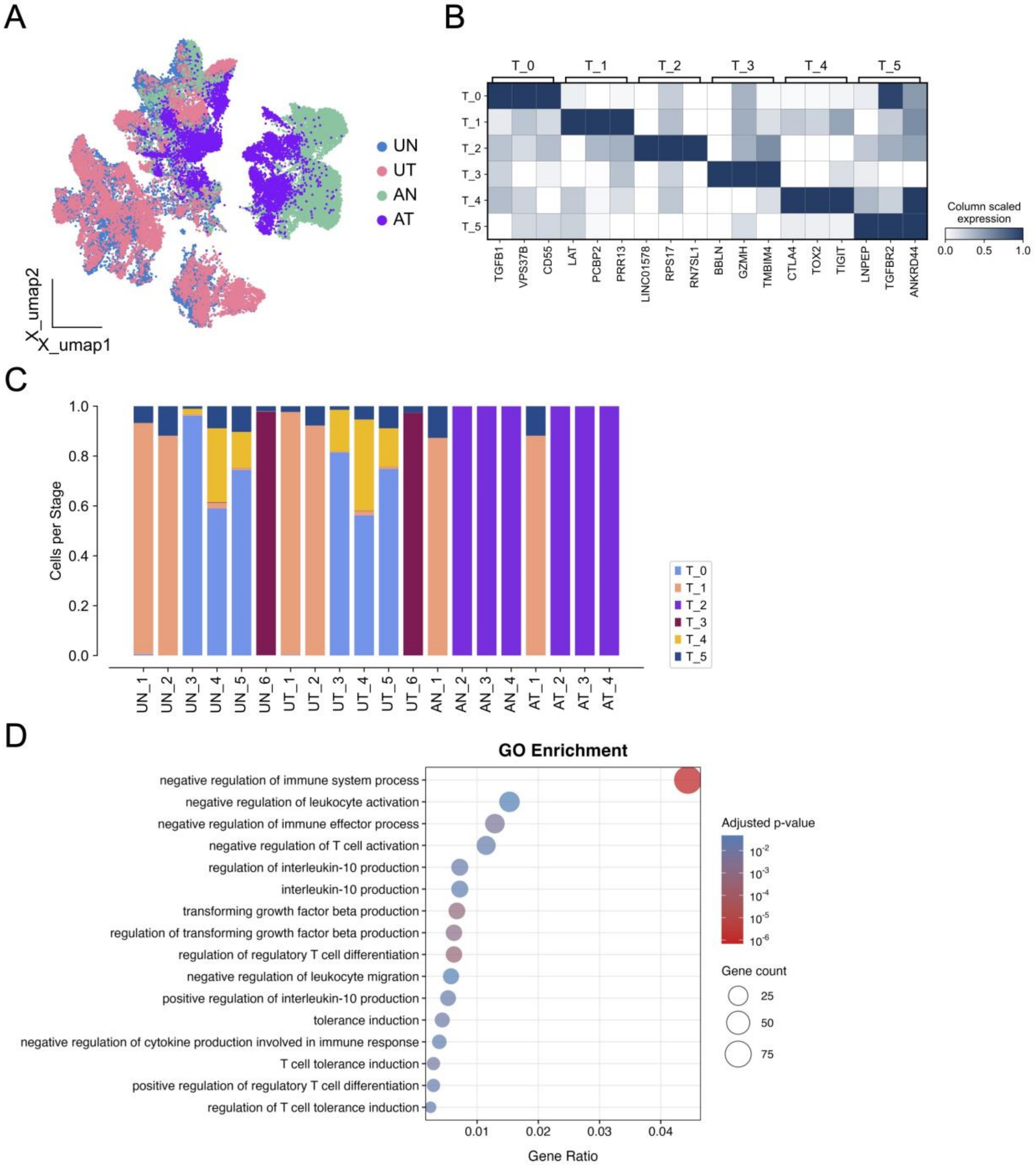

**Supplementary Figure 3.** Related analyses of epithelial subsets. (A-B) UMAP plots of epithelial clusters and their distribution across clinical groups. (C) Proportional distribution of subsets across individual samples. (D-E) Heatmap and dot plot showing marker expression across subsets. (F) KEGG enrichment highlighting functional specialization of epithelial subsets.

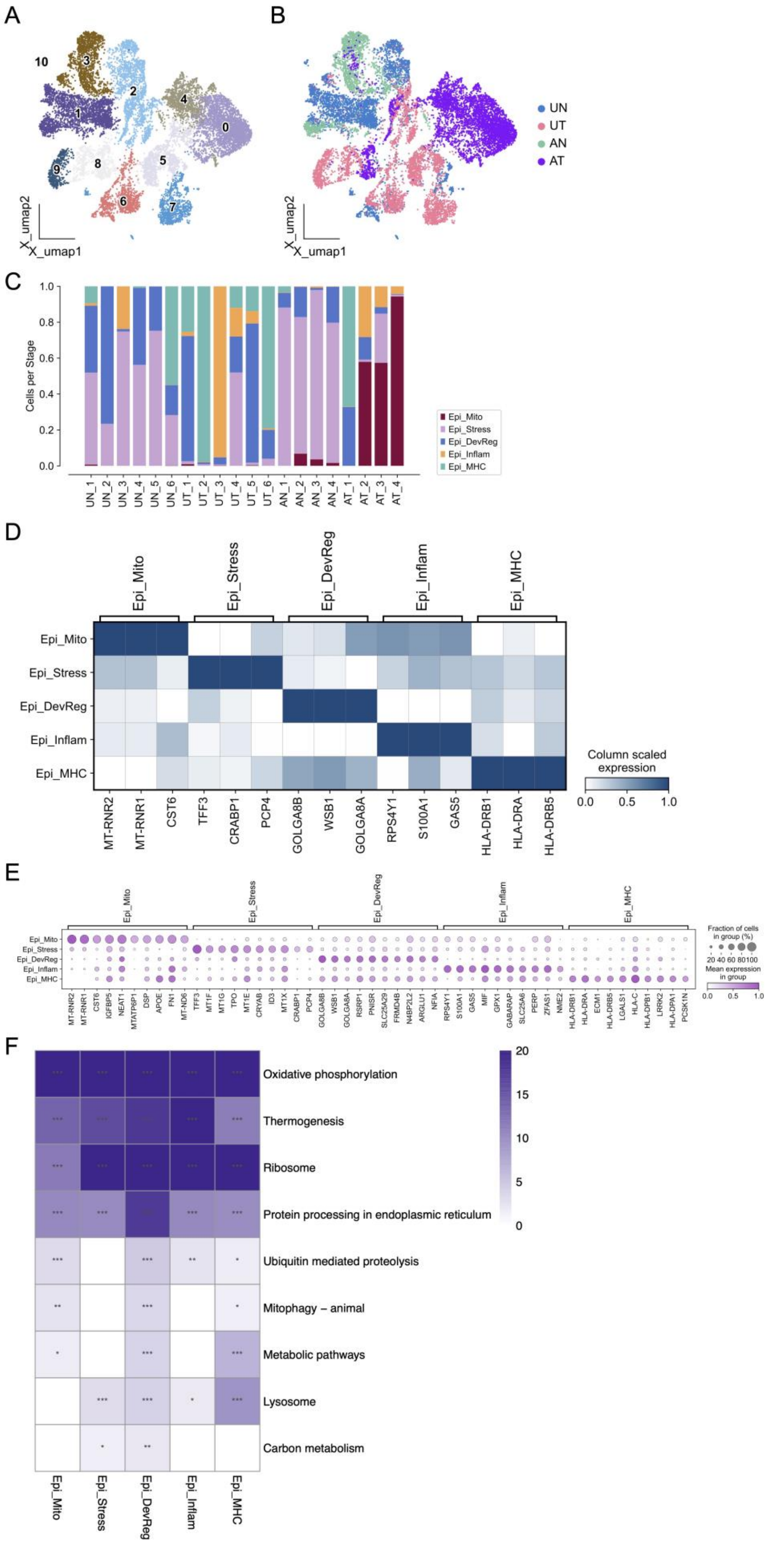
A
10
3
2
4
1
0
9
8
5
6
7
X_umap2
X_umap1
B
UN
UT
AN
AT
X_umap2
X_umap1
C
Cells per Stage
1.0
0.8
0.6
0.4
0.2
0.0
UN_1
UN_2
UN_3
UN_4
UN_5
UN_6
UT_1
UT_2
UT_3
UT_4
UT_5
UT_6
AN_1
AN_2
AN_3
AN_4
AT_1
AT_2
AT_3
AT_4
Epi_Mito
Epi_Stress
Epi_DevReg
Epi_Inflam
Epi_MHC
D
Epi_Mito
Epi_Stress
Epi_DevReg
Epi_Inflam
Epi_MHC
Column scaled expression
0.0
0.5
1.0
MT-RNR2
MT-RNR1
CST6
TFF3
CRABP1
PCP4
GOLGA8B
WSB1
GOLGA8A
RPS4Y1
S100A1
GAS5
HLA-DRB1
HLA-DRA
HLA-DRB5
E
Epi_Mito
Epi_Stress
Epi_DevReg
Epi_Inflam
Epi_MHC
Fraction of cells in group (%)
20 40 60 80 100
Mean expression in group
0.0 0.5 1.0
F
20
15
10
5
0
Oxidative phosphorylation
Thermogenesis
Ribosome
Protein processing in endoplasmic reticulum
Ubiquitin mediated proteolysis
Mitophagy – animal
Metabolic pathways
Lysosome
Carbon metabolism
Epi_Mito
Epi_Stress
Epi_DevReg
Epi_Inflam
Epi_MHC

**Supplementary Figure 4.** Validation of TAM subtypes across thyroid cancer samples.

(A) Cross-sample integration confirming reproducibility of TAM subtype identification.

(B) Heatmap showing subset-specific marker expression across patients.

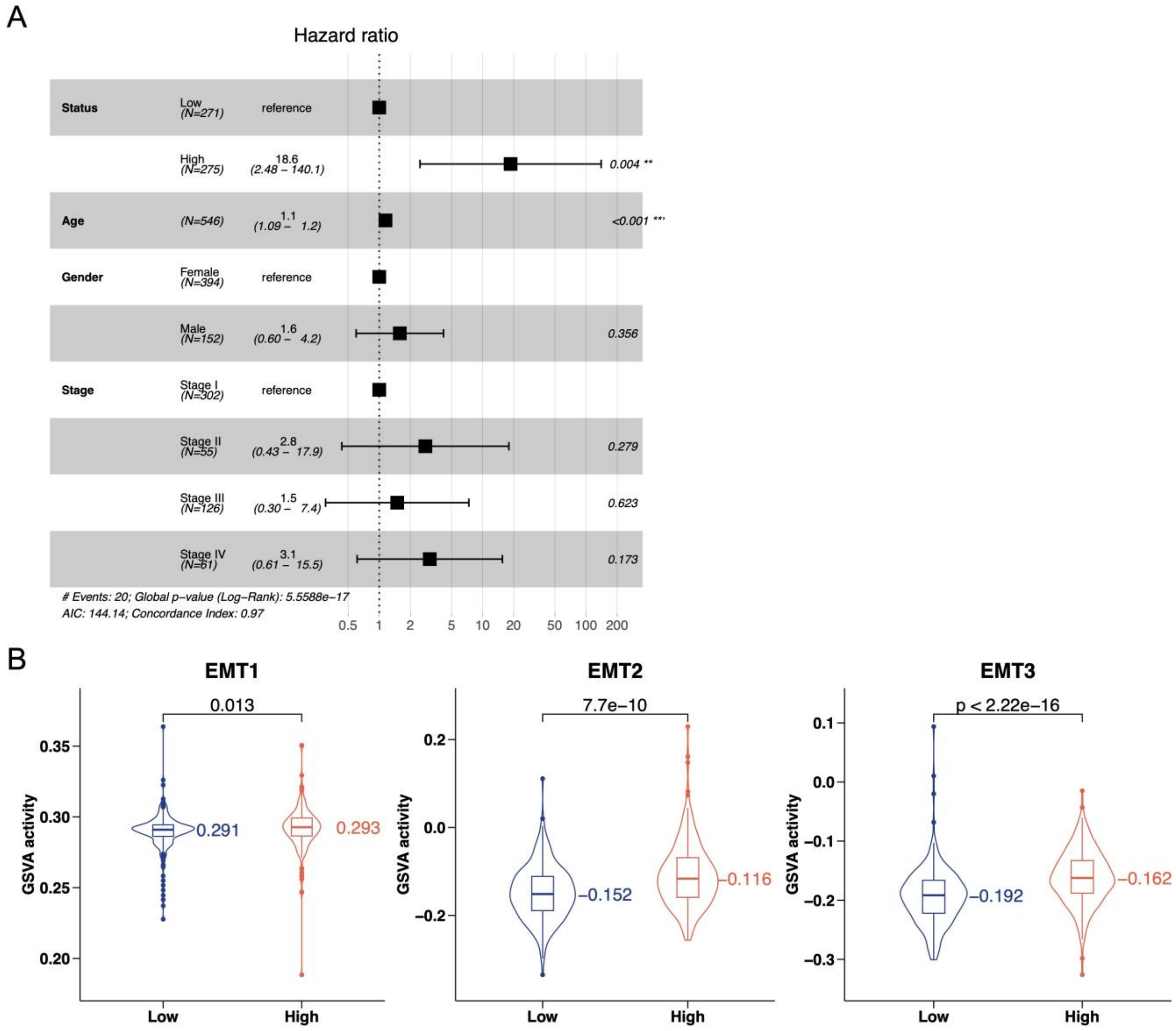

**Supplementary Figure 5.** EMT-related transcriptional reprogramming in thyroid cancer. (A-B) Boxplots showing increased expression of EMT regulators (*TGFB1*, *PHTF2*, *SNAI1*, *SLUG*, *TWIST1/2*, *ZEB1/2*) in high-signature tumors. (C) ESTIMATE score and tumor purity comparison between low- and high-signature groups.

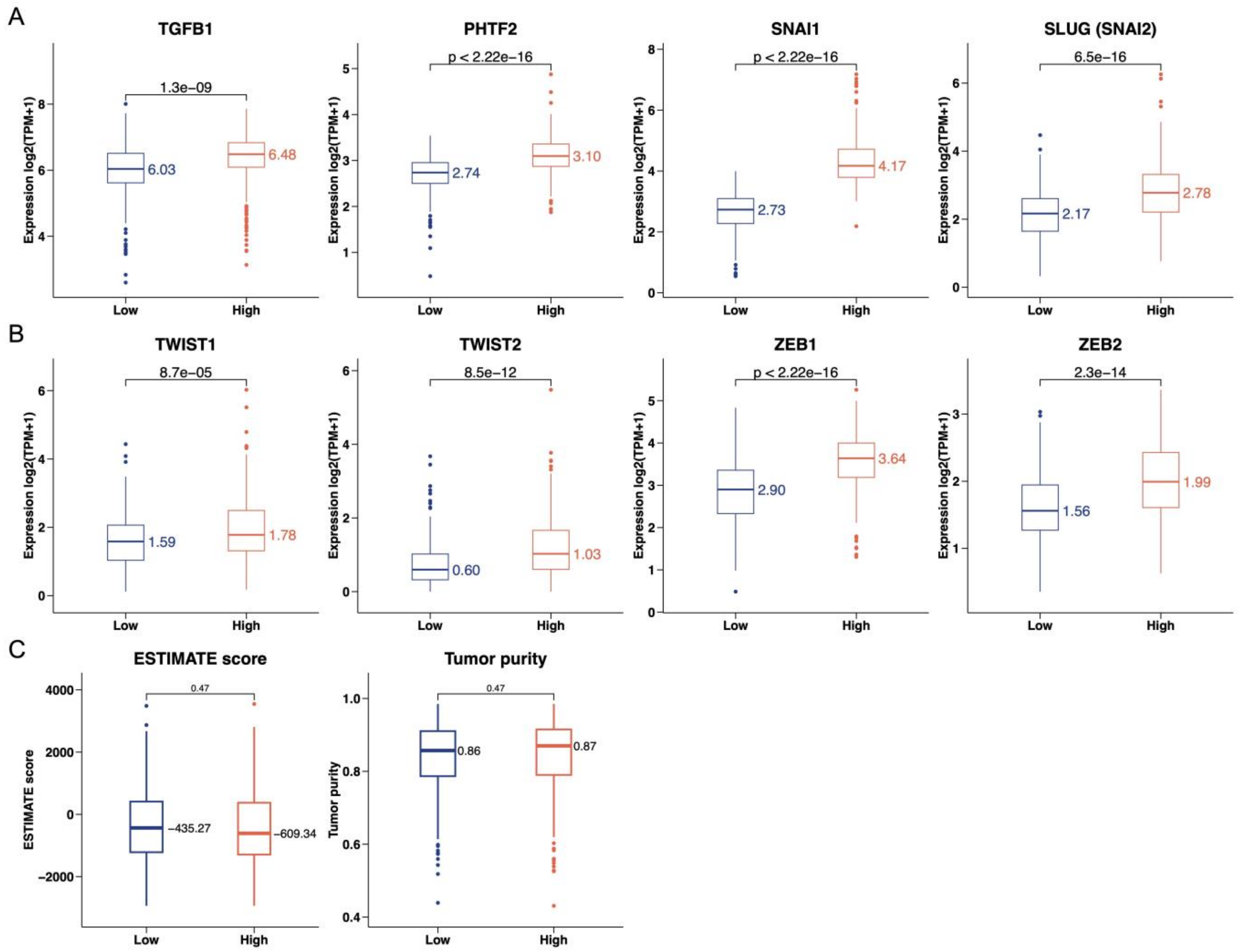

Supplement: Supplementary file 1 [file supplementary_figures.pdf]
